# Challenges in diagnosis and management of neonatal hyperparathyroidism in a resource-limited country: a case series from a Sudanese family

**DOI:** 10.11604/pamj.2021.40.105.29527

**Published:** 2021-10-15

**Authors:** Samar Sabir Hassan, Marlies Kempers, Dorien Lugtenberg, Asmahan Tajelsir Abdallah, Salwa Abdelbagi Musa, Areej Ahmed Ibrahim, Mohamed Ahmed Abdullah

**Affiliations:** 1 Department of Pediatric Endocrinology, Gaafar Ibn Auf Pediatric Tertiary Hospital, Khartoum, Sudan,; 2 Radbound University Medical Center, Nijmegen, Netherlands,; 3 Sudan Childhood Diabetes Center, Khartoum, Sudan,; 4 Faculty of Medicine, University of Khartoum, Khartoum, Sudan

**Keywords:** Neonatal hyperparathyroidism, calcium sensing receptor, parathyroidectomy, hypercalcemia

## Abstract

Neonatal hyperparathyroidism is a rare disease caused by a homozygous inactivating mutation in the calcium sensing receptor gene. It presents early in life with life threatening manifestations of hypercalcemia, if left untreated the condition may be lethal. This is the first case series reported from Sudan. Three Sudanese siblings presented with severe symptoms of hypercalcemia in the form of polyuria, failure to thrive and multiple bone fractures. Serum calcium and parathyroid hormone levels were very high with low phosphate and normal alkaline phosphatase levels. Ultrasonography and sestamibi scan were normal and did not assist in diagnosing their condition. Medical management was a great challenge due to unavailability of medications such as parentral bisphosphonates and calcimimetics. Parathyroidectomy was inevitable. Tissue biopsies revealed parathyroid hyperplasia and no adenoma. Gene sequencing revealed a homozygous missense mutation: c 2038 C T p (Arg680Cys) in two siblings, both parents were heterozygous for the same missense mutation. Our report reflects the challenges in diagnosis and management of neonatal hyperparathyroidism in resource limited countries. We also highlight the importance of genetic testing in the diagnosis and management of such cases in countries with high rates of consanguineous marriage.

## Introduction

The calcium sensing receptor (CaSR) plays a crucial role in calcium homeostasis. The human CaSR gene (Online Mendelian Inheritance in Man (OMIM #601199) is located at chromosome 3q13.3-q21.1) [1]. More than 100 mutations in the CaSR gene are known to date. Loss of functional mutations results in hypercalcemia with hypocalciuria. There are two forms, a heterozygous mutation resulting in a benign asymptomatic form not requiring treatment, called familial benign hypocalciuric hypercalcemia (FBHH) (OMIM #145980) typically characterized by moderate elevations of serum calcium concentration, inappropriately low urinary calcium excretion, and high normal or mildly elevated parathyroid hormone (PTH) levels [2-5]. The other being a homozygous mutation resulting in a more rare but severe form called neonatal hyperparathyroidism (NHPT) (OMIM # 239200) requiring urgent treatment. The latter NHPT presents in the first 6 months of life with severe manifestations of hypercalcemia in the form of respiratory, skeletal, and psychomotor symptoms. PTH levels are high, and if left untreated the condition may be lethal [2-5]. We here report, and for the first time from Sudan, three siblings with this rare condition and discuss the problems that we faced in diagnosing and managing this disorder in a resource-limited country.

## Methods

**Figure 1:**
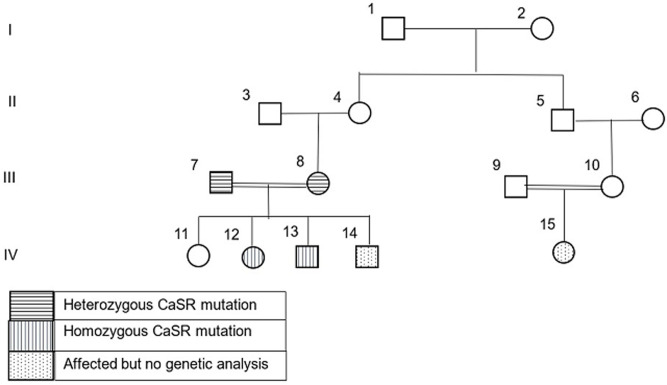
family pedigree

Case 1 {(1V/12), Figure 1}: a 7-month-old female presented with multiple bone fractures and was accidentally found to have hypercalcemia on routine investigations. She had a history of NICU admission for what was thought to be neonatal sepsis because of poor feeding and floppiness, chronic constipation, and failure to thrive but no history of urinary stones. Her birth was 2.8 kg. There were no dysmorphic features and her weight at presentation was 5 kg (-2.6 standard deviation (SD) below the mean for her age and sex). Parents are healthy first-degree cousins. The daughter of her maternal cousin was diagnosed with hyperparathyroidism in infancy which required parathyroidectomy. The biochemical, radiological, and histopathological findings are shown in Table 1. Her mother´s calcium level was 11.3 mg/dl (2.83 mmol/l), father´s level was 9.1 mg/dl (2.27 mmol/l). Vitamin D3 was not measured for the parents. She only showed a partial response to medical treatment of hypercalcemia, in the form of intravenous saline with furosemide 2 mg/kg/dose TDS, bisphosphonates in the form of oral alendronate 10 mg/day and calcitonin 4 IU/kg given subcutaneously every 6 hours. The lowest calcium level achieved with medical treatment was 17 mg/dl. Surgical intervention was inevitable and included total parathyroidectomy through bilateral neck incision and removal of the four parathyroid glands with re-implantation of one gland in the sternocleidomastoid muscle. Following surgery, she remained symptomatic with persistently high levels of calcium and PTH. The condition was not responsive to same medical treatment mentioned and required a second surgery to remove any remaining parathyroid tissue 8 weeks following the first surgery. Her calcium levels normalized immediately following the second surgery and after which she remained asymptomatic with normal calcium levels without any treatment. She managed to catch up normal growth for her age by the age of 36 months.

**Table 1:** biochemical, radiological and histopathological findings in case one and two

Investigation	Case 1	Case 2	Normal values
Serum calcium mg/dl (mmol/L)	22.5 (5.5)	19.5 (4.86)	8.5 - 10.2 (2.1 - 2.6)
Serum phosphorus mg/dl (mmol/L)	1.4 (0.452)	1.1 (0.35)	2.5 - 4.5 (1.12 - 1.4)
Alkaline phosphatase IU/L	178	235	150 - 420
PTH pg/ml	229	838.5	10-55
UCCR mol/mol	0.026	0.024	0.09 - 2.2
Vitamin D3 ng/ml	38	Not done	≥30
X-ray of long bones	Osteopenia, multiple bone fractures	Osteopenia	-
Ultrasonography of parathyroid glands	Enlarged parathyroid glands	Enlarged parathyroid glands suggestive parathyroid adenoma	-
SestaMibi scan	Not done	Normal uptake scan	-
Histopathology of parathyroid gland	Chief cell hyperplasia, water clear cell hyperplasia, no adipose tissue	Chief cell hyperplasia, water clear cell hyperplasia, no adipose tissue	-

PTH: parathyroid hormone; UCCR: urinary calcium creatinine ratio

Case 2 {(1V/13),(Figure 1)} a younger male sibling, presented at the age of 8 months with a history of chronic constipation, abdominal pain, polyuria, failure to thrive, and floppiness since birth. a younger male sibling, presented at the age of 8 months with a history of chronic constipation, abdominal pain, polyuria, failure to thrive, and floppiness since birth. He had no history of bone fractures nor urinary stones. There were no dysmorphic features and his weight was 6 kg (-3.5 SD). His biochemical and radiological findings are shown in Table 1. Hypercalcemia was resistant to available medical treatment in the form of intravenous saline and furosemide, oral bisphosphonates, and calcitonin. Surgical intervention was required and removal of all four parathyroid glands with reimplantation of the fourth one in the sternocleidomastoid muscle was done. Immediately following surgery, he developed hypocalcemia and required intravenous calcium then was shifted to oral calcium supplementations, 40 mg/kg/day for 6 weeks after which his calcium levels normalized. There after 6 weeks from his surgery he remained asymptomatic with normal calcium levels and required no further treatment. He managed to catch up to normal growth for his age at 30 months.

Case 3 {(1V/14), (Figure 1)}: the younger sibling presented post-delivery with severe symptoms of hypercalcemia, bone fractures and respiratory distress that required ventilatory support. PTH and calcium levels were high. Management required parathyroidectomy. Further medical data was difficult to access as this patient was born abroad in Saudi Arabia (Figure 1).

## Results

**Figure 2:**
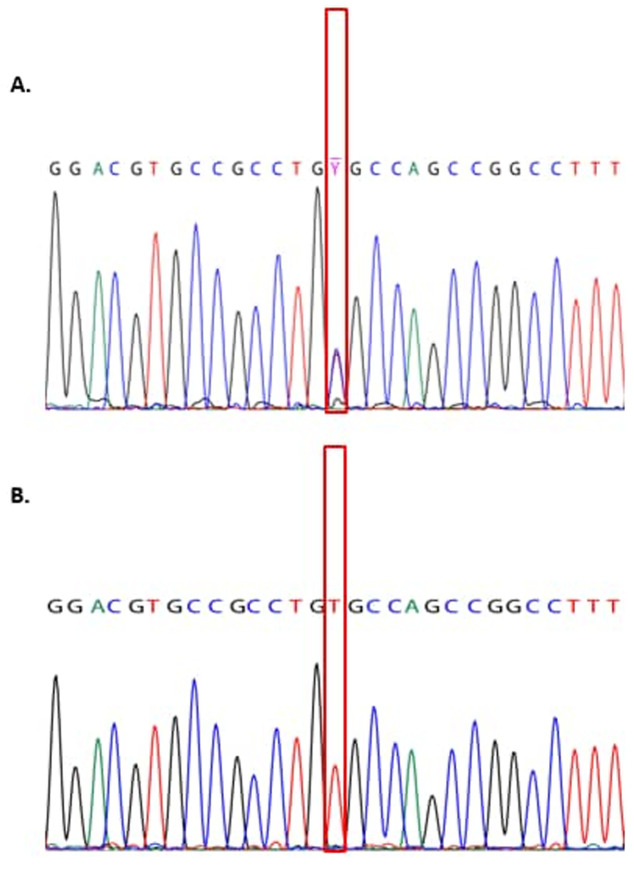
chromatogram of CaSR gene mutation in family members

Biochemical and radiological findings are shown in (Table 1). Genetic analysis (Figure 2): genetic testing polymerase chain reaction (PCR) products covering all coding regions and splice sites of the CaSR gene were amplified using genomic DNA. After purification, Sanger sequencing was performed on an applied biosystem 3730 automated sequencer. Primer sequences are available upon request.

In two affected siblings case 1 (1V/12), and case 2 (1V/13) shows a homozygous pathogenic mutation in the CaSR gene (NM_00388.3) which was detected by DNA sequence analysis. It is a missense mutation: c. 2038C ≥ T (p. (Arg680Cys)). Both parents were found to be heterozygous for the same missense mutation. No available genetic data for case 3 (1V/14), and no mutation was identified in the non-affected sister (Figure 1). The mutation has been described [5] and functional testing has proven pathogenicity [6-8].

## Discussion

NHPT is a rare condition and only a few cases including ours were reported in the literature to date [9-12]. To the best of our knowledge, this is the first reported cases from Sudan and sub-Saharan Africa. Sudan is a country with high rates of consanguineous marriage. In this report three infants were affected from one family. Apart from bone fractures and respiratory distress, symptoms of hypercalcemia in infants can be nonspecific and therefore diagnosis can be delayed as happened to our cases. Even if hypercalcemia is diagnosed, in developing countries, there may be difficulties in identifying the cause due to lack of facilities for PTH assay in primary and secondary care setups. Limited access to medical data on case {(1V/14), (Figure 1)} made it difficult to compare his management and diagnosis to his two elder siblings. Nevertheless, availability of facilities and medications assisted in early diagnosis and management of this case in comparison to his elder two siblings who were born in Sudan.

Inactivating mutations in CaSR may affect a single allele (heterozygosis), resulting in the phenotype characteristic of FBHH, or both alleles (homozygosis or compound heterozygosis if no consanguinity exists), leading to NHPT, so that the degree of gene defect is responsible for the great difference in the phenotypic presentation [2,3]. In this report, two siblings had NHPT with homozygous mutations and variability in the degree of severity of symptoms In case 1 {(1V/12), (Figure 1)} the symptoms were severe in the face of high, but not too high PTH and extremely high calcium levels, while her younger sibling, case 2 {(1V/13), (Figure 1)} presented with milder symptoms, high calcium levels and extremely high levels of PTH. and extremely high calcium levels, while her younger sibling, case 2 {(1V/13), (Figure 1) presented with milder symptoms, high calcium levels and extremely high levels of PTH. These findings suggest no correlation between the levels of PTH, calcium and the severity of clinical symptoms. NHPT shows a puzzling range of serum calcium and PTH levels. In a recently published study, the levels of serum calcium and PTH were compared between patients with NHPT with homozygous mutations versus those with heterozygous mutations. They concluded that homozygotes for pathogenic CaSR variants show higher calcium and PTH levels than heterozygotes and calcium levels above 4.5 mmol/L among NHPT are frequent and unique only to most homozygotes [13]. Zajickova *et al*. reported that vitamin D levels may play an important role in the degree of severity of hypercalcemia in a patient with CaSR mutation, be it either the patient´s own vitamin D level or the lack of vitamin D in the mother during pregnancy [14]. Their patient with low 25 OH vitamin D3 levels benefited from vitamin D supplementation. In our report, 25 OH vitamin D3 level was normal in case 1 {(1V/12), (Figure 1)} and was not measured in case 2 {(1V/13), (Figure 1)} or their parents because of the cost. Typically, cases of CaSR inactivation mutation present with hypocalciuric hypercalcemia. A clinical finding which characterizes it thus helps distinguish them from primary hyperparathyroidism. In contrary to adults, cut of levels for diagnosing hypocalciuria in children vary with age making it more difficult to define the cut off levels in children. In comparison to a study done by Matos *et al*. [15] two of our patients showed an inappropriately low urinary calcium creatinine ratio (UCCR).

In addition to the clinical picture, genetic studies are very useful. However, this is not available in developing countries and even if done abroad it is received late as was in our case, and therefore management including parathyroidectomy can be done based on clinical grounds. Medical emergency management should be initiated by the restoration of extravascular volume followed by furosemide. Calcitonin given subcutaneously can also give some short-term improvement in serum calcium. Calcitonin was not initially available in our setting and was sent to the family from relatives living abroad. Bisphosphonates are second line treatment they act by halting bone resorption. In two separate studies Al-Shafaney and Waller, reported the benefit of bisphosphonates in managing mild cases and helping to bridge the severe cases for surgery [9,16]. Parenteral bisphosphonates are better than oral forms however, unlike the situation now, they were not initially available to us. Nevertheless, the oral bisphosphonates that we used in our patients gave temporary partial suppression of calcium level even better than calcitonin. Cinacalcet is known to increase the sensitivity of CASR [14] and is reported to be effective in heterozygous mutation causing NHPT on the contrary to homozygous cases where four reported heterozygous cases did not require parathyroidectomy [17]. Unfortunately, parenteral bisphosphonates and cinacalcet were not available to us at that time which made managing such cases extremely challenging. Therefore, parathyroidectomy was considered before receiving the results of genetic studies.

Pre-operative imaging using ultrasonography or magnetic resonance or sestamibi scan and or intraoperative measurements of PTH levels may help guide the extent of parathyroid resection, particularly in the case of multigland hyperparathyroidism but these imaging studies do not usually help in children with neonatal and familial hyperparathyroidism [11]. Al-Shanafey *et al*. published series of five cases with NHPT; all patients had the ultrasound, computed tomography (CT) scan and sestamibi nuclear scan but these studies could not identify the parathyroid glands in any of them [9]. In many developing countries radio nuclear imaging may not be available and neonatal ultrasonography needs professional experience. In our report preoperative imaging using sestamibi scan was highly expensive and not helpful and the ultrasonography gave a false impression of parathyroid adenoma.

Surgical intervention is inevitable in patients with severe clinical manifestations of hypercalcemia not responding to medical therapy. Surgery in infants is quite challenging owing to the difficulty in parathyroid gland identification, requiring sufficient expertise. Nowadays, total parathyroidectomy with or without autotransplantation is increasingly being performed in most centers [9-12]. Parathyroidectomy could be subtotal or total with - implantation of one gland in the sternocleidomastoid muscle of the non-dominant arm [18,19]. In cases of NHPT, usually, parathyroidectomy of all 4 glands is required to achieve a cure and less radical removal may result in the persistence of hyperparathyroidism and hypercalcemia [9,11,19]. Some surgeons recommend parathyroid auto transplantation, yet frequently these grafts fail to work [9,19]. It has been reported that autotransplantation may lead to graft dependent hypercalcemia in 33% and a failure rate of 6% [9-11,20]. Al Shafaney *et al*. reported no recurrence following autotransplantation in his series of five patients [9]. Alagaratnam *et al*. reported in a series of cases with one case who remained moderately hypercalcemic with elevated PTH following subtotal parathyroidectomy and was managed conservatively [11]. Savas-Erdeve *et al*. have reported a single case of recurrence following autotransplantation which required removal [10]. The latter study resembling our first patient who required two surgeries to become symptom free and normalize her calcium levels.

Failure of surgery to cure hyperparathyroidism may be due to either multi gland disease (e.g. hyperplasia) unidentified by preoperative imaging studies or failure to locate parathyroid glands in unusual areas (ectopic glands). In case {(1V/12), (Figure 1)} reimplantation was not successful. Postoperative symptomatic hypocalcemia is a common postoperative complication. Alagaratnam *et al*. reported in a series of cases, five cases requiring hypocalcemia treatment following total parathyroidectomy [11] as happened to case 2 {(1V/13), (Figure 1)}. Various operative adjuncts such as intra-operative PTH monitoring, radio-guided parathyroidectomy, frozen section, and methylene blue are commonly used to overcome this problem and improve the cure rate. Unfortunately, these techniques are unavailable in our settings. Hence, the best operative option for cases of severe NHPT in places similar to our setting is total parathyroidectomy without reimplantation.

## Conclusion

Neonatal hyperparathyroidism (NHPT) is a rare and challenging disorder and dynamic management strategies are highly required. Hence, management of such cases is extremely challenging in resource limited countries. Genetic testing is of great value in populations with high rates of consanguineous marriage like Sudan.

**Limitations:** i) limited resources in our setting contributed to the lack of ability to perform some investigations and give the standard methods of therapy; ii) limited access to medical data on case 3 made it difficult to compare his management and diagnosis to his two elder siblings.

### What is known about this topic

- Neonatal hyperparathyroidism is a rare disease caused by a homozygous inactivating mutation in the calcium sensing receptor gene; it presents early in life with life threatening manifestations of hypercalcemia, if left untreated the condition may be lethal.

### What this study adds

- To our knowledge this is the first case series report of neonatal sever hyperparathyroidism from Sudan, an African country with high rate of consanguineous marriage;
- Our findings suggest no correlation between the levels of parathyroid hormone and serum calcium levels and the severity of clinical symptoms in neonatal severe hyperparathyroidism;
- Although genetic testing is highly helpful in cases neonatal severe hyperparathyroidism, yet in developing countries where it is not available, management including parathyroidectomy can be done based on clinical grounds; findings in our study recommend that the best operative option for cases of neonatal severe hyperparathyroidism in places with limited resources similar to our setting is total parathyroidectomy without reimplantation.
